# Changes to the reproductive microbiome of the brood pouch during male pregnancy in seahorses (*Hippocampus abdominalis*)

**DOI:** 10.1530/REP-24-0159

**Published:** 2025-03-10

**Authors:** Jenny Wang, Zoe M G Skalkos, Catherine E Grueber, Camilla M Whittington

**Affiliations:** School of Life and Environmental Sciences, The University of Sydney, Sydney, New South Wales, Australia

**Keywords:** brooding, gestation, microbial community, placenta

## Abstract

**In brief:**

Reproductive microbiomes contribute to the successful embryogenesis of offspring but are poorly studied in non-mammalian species that exhibit pregnancy. This study characterises the male pregnant seahorse brood pouch microbiome and identifies potential microbial maternal contributions to the pouch, providing insights into the sources and adaptive value of the embryonic microbial environment.

**Abstract:**

Seahorses demonstrate an unusual reproductive strategy, in which males incubate embryos inside a complex ‘brood pouch’ until parturition, analogous to mammalian viviparity. In many species, a ‘normal’ reproductive microbiome ensures successful embryogenesis and enables parents (usually mothers) to provide their offspring with their initial microbiome. In male-pregnant seahorses, embryos may receive microbiomes from both parents: from the paternal brood pouch and from the maternal eggs. Using the pot-bellied seahorse (*Hippocampus abdominalis*), we employed 16S rRNA sequencing to explore the reproductive microbiome. We aimed to compare the microbiome of the male pregnant pouch to the male non-pregnant pouch and external skin, and to identify bacterial taxa found exclusively in the pregnant pouch that could be derived maternally from the microbiome of eggs. Our findings demonstrate that the pregnant brood pouch microbiome is compositionally distinct from the non-pregnant pouch and external skin. The pouch microbiome also has characteristics of resistance to colonisation by pathogens, including a low species richness, high species evenness and diversity and very low abundance of *Vibrio*, a genus that includes fish skin pathogens. Thirteen bacterial taxa appear exclusively in the pregnant pouch, relative to the non-pregnant pouch, and seven of these overlapped with taxa present in or on the eggs. The possible supplementation of brood pouch microbiome with egg-associated micro-organisms hints at a maternal microbial contribution to male pregnancy. This characterisation of the pregnant seahorse pouch microbiome provides a platform for further research into its function and possible adaptive value during male pregnancy.

## Introduction

Syngnathids (seahorses, pipefish and seadragons) demonstrate advanced parental care through brooding (parental incubation of eggs in or on the body, outside the female reproductive tract), a process analogous to mammalian gestation that is termed ‘male pregnancy’ (Whittington & Friesen 2020). Male seahorses have the most complex brooding anatomy in Syngnathidae (Whittington & Friesen 2020, Harada *et al.* 2022). Their highly specialised brood pouches incubate embryos until their release as free-swimming neonates (Carcupino *et al.* 2002). The seahorse pouch is a physiologically modified extension of the tail skin that opens at a single pore (Kawaguchi *et al.* 2017). Made up of a thick, vascularised dermis containing a placental layer, it facilitates physiological exchange between fathers and embryos during pregnancy (Whittington & Friesen 2020, Whittington *et al.* 2022). The pouch tissue changes morphologically and physiologically during pregnancy and facilitates respiratory gas exchange (Dudley *et al.* 2021), nutrient transfer (Skalkos *et al.* 2020, 2024) and putatively waste removal and immunological protection (Melamed *et al.* 2005, Roth *et al.* 2012, Whittington *et al.* 2015).

During mating, the seahorse brood pouch fills with non-sterile seawater, and the addition of nutrient-rich eggs, some of which break, likely produces an environment conducive to microbial proliferation (Whittington & Friesen 2020, Zhao *et al.* 2023). The male pouch microbiome may play a vital role in pregnancy and embryo development akin to gestational microbiomes in classical female viviparity. In viviparous animals, reproductive tract-associated microbiomes directly influence host reproductive success, as disruptions to the normal microbiome (dysbiosis) can harm offspring (Comizzoli *et al.* 2021, Rowe *et al.* 2021). Commensal micro-organisms can defend against pathogens by producing antimicrobial agents and via competitive exclusion (Tachedjian *et al.* 2017, Rowe *et al.* 2021). Reproductive microbiomes also facilitate vertical transmission, the inheritance of micro-organisms from mother to offspring (Funkhouser & Bordenstein 2013). Vertical transmission can immunologically benefit offspring by limiting pathogen invasion (Kerwin *et al.* 2019, Bunker *et al.* 2021), seeding commensal micro-organisms (Mika *et al.* 2021) and priming the embryonic immune system (Beemelmanns *et al.* 2019). In seahorses, the pouch microbiome may similarly protect embryos and enable vertical transmission.

In addition to acquisition of micro-organisms from direct contact with the male pouch microbiome, eggs may act as vessels for maternal vertical transmission to offspring. Thus, the seahorse gestational microbiome could be plausibly sourced both paternally and maternally. Research on egg-mediated microbial transfer primarily focuses on oviparous animals with limited parental care (Funkhouser & Bordenstein 2013, Nyholm 2020). In oviparous species, commensal micro-organisms in or on eggs can protect offspring against pathogenic fouling (Liu *et al.* 2014, Kerwin *et al.* 2019, Bunker *et al.* 2021). Beneficial micro-organisms are packaged inside eggs and/or inoculate their surface for maternal germline transmission (Cary & Giovannoni 1993, Hosokawa *et al.* 2013, Van Veelen *et al.* 2018, Bunker *et al.* 2021). Micro-organisms contained inside eggs are absorbed by developing embryos while egg surface micro-organisms are acquired after hatching (Nyholm 2020). A study in broad-nosed pipefish (*Syngnathus typhle*) demonstrated that micro-organisms inside eggs colonise the offspring gut (Tanger *et al.* 2024). Therefore, both the female egg and male pouch microbiome warrant investigation as potential sources of embryonic micro-organisms.

While the non-pregnant brood pouch microbiome has been characterised in lined seahorses (*Hippocampus erectus*) (Zhao *et al.* 2023), the pregnant seahorse pouch microbiome and its potential protective role for embryos remain unexplored. Previous studies in pipefish have examined the egg microbiome and its contributions to embryos (Beemelmanns *et al.* 2019, Tanger *et al.* 2024), but we lack such investigations in seahorses, which have more complex, closed pouches that permit fathers more control over the embryonic environment (Whittington & Friesen 2020).

We used the pot-bellied seahorse (*Hippocampus abdominalis*) to address two main questions regarding the sources of the seahorse gestational microbiome. First, does the male pregnant pouch harbour a unique microbiome compared to the non-pregnant pouch and external skin? Second, are there micro-organisms exclusive to the pregnant pouch that are not otherwise found in the non-pregnant pouch, and are these taxa on and/or in female eggs? For our first aim, we characterised the male pregnant pouch by assessing bacterial species richness, diversity, community structure and taxonomic composition, comparing it to the male non-pregnant pouch and external skin. For our second aim, we identified micro-organisms unique to the male pouch and assessed their overlap with the taxonomic profile of the female eggs to identify any potential egg-derived taxa.

## Methods

### Animal husbandry and breeding

Captive bred, reproductively mature *H*. *abdominalis* were obtained from Seahorse Australia (TAS, Australia) (University of Sydney Animal Ethics Committee; approval number: 2021/1995). Animals were housed in recirculating aquaria under standard conditions, including artificial seawater, at The University of Sydney (Australia), as previously described (Whittington *et al.* 2013). Tanks were cleaned twice weekly, during which water quality parameters were monitored. The water used in the aquaria was prepared in a controlled laboratory environment, which may influence the microbiome differently than natural environments. Animals were fed thawed frozen *Mysis relicta* shrimp (Ocean Nutrition, USA) six days each week and held under a summer light regime (15.5h light:8.5h darkness) to stimulate natural breeding behaviours. Males were tagged with coloured bead ‘necklaces’ for identification. To obtain pregnant males, we co-housed four non-pregnant males and five females in a deep 750 L breeding tank for three-day periods (Woods 2000). After mating, males were transferred to 170 L shallow-water tanks, separated from females. To determine which males were pregnant after three days in the breeding tank, we assessed their reproductive status using established behavioural testing methods (Whittington *et al.* 2013). We conducted behavioural trials between 08 30 and 10 30 h, when seahorses are most reproductively active, over three consecutive days (Masonjones & Lewis 1996, Whittington *et al.* 2013). We repeated this breeding and behavioural testing cycle until we obtained five putatively pregnant males. We housed putatively pregnant males separately from non-pregnant males until mid-pregnancy (14–17 days post-fertilisation), roughly corresponding to 70% of embryonic development (Sommer *et al.* 2012).

### Sample collection

Animals were euthanised by gradual overdose of ethanol, followed by decapitation and pithing, in accordance with protocols approved by the University of Sydney Animal Ethics Committee (approval number: 2021/1995) and the American Veterinary Medical Association 2020 (Leary *et al.* 2020). Microbial samples were collected using sterile flocked swabs (Rongye Technology, China) and stored immediately at −20 °C until DNA extraction.

**Figure 1:**
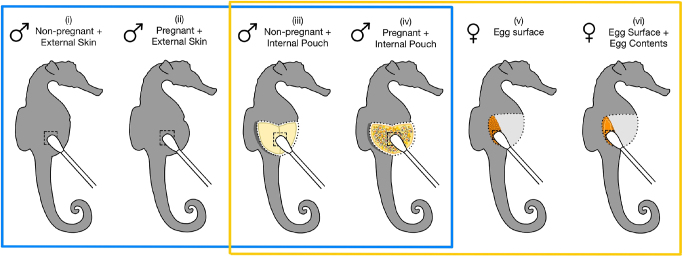
Diagram of swab samples collected for microbiome analysis from male and female *Hippocampus abdominalis* adults. (i) Non-pregnant external skin, outside of pouch (male). (ii) Pregnant external skin, outside of pouch (male). (iii) Non-pregnant internal pouch (male). (iv) Pregnant internal pouch containing mid-stage embryos (14–17 days post-fertilisation) (male). (v) Ovary with intact eggs (female). (vi) Ovary with punctured eggs (female). Blue box indicates experimental groups used to address Aim 1 (comparison of male non-pregnant and pregnant external skin and internal pouch microbiomes). Yellow box indicates experimental groups used to address Aim 2 (to identify bacterial taxa exclusive to the male pregnant pouch and assess their presence on and/or in female eggs). Dashed line indicates swabbing area and dotted line indicates where incisions were made to dissect animals.

We collected two microbiome samples (repeated measures) from each male: one external skin surface swab (skin outside of the pouch) and one internal brood pouch swab (Fig. 1i, ii, iii, iv). We took the first sample by swabbing the skin surface of each male. We then opened brood pouches by making an incision with a sterile razor blade from the pouch opening to the base. We took the second sample by swabbing the brood pouch tissue surface (non-pregnant males) or the surface of the pouch and interspersed embryos (pregnant males 14–17 days post-fertilisation). In both cases, we passed the swab head 10 times over a ∼1 cm^2^ area. We collected 20 microbiome samples in total from four experimental groups: i) non-pregnant external skin samples and iii) non-pregnant internal pouch (*n* = 5 non-pregnant males), and ii) pregnant external skin samples and iv) pregnant internal pouch (*n* = 5 pregnant males) (Fig. 1i, ii, iii, iv).

We collected two microbiome samples (repeated measures) from each female: one egg surface swab and one egg surface plus egg contents swab (Fig. 1v and vi). We opened the abdominal cavity of each female and cut the membrane of one randomly selected ovary using sterile surgical scissors to expose the intact eggs. We took the first sample by swabbing the external surface of the eggs. We took the second sample by puncturing the egg membranes using sterile surgical scissors and swabbing the released internal contents. For all female samples, we passed the swab head 10 times over a ∼0.5 cm^2^ area (due to the size constraints of the ovary). We collected ten microbiome samples in total from two experimental groups: v) egg surface samples and vi) egg surface plus egg contents (*n* = 5 females) (Fig. 1v and vi).

### DNA extraction, amplification and sequencing

We extracted DNA from swab samples using the DNeasy Blood and Tissue Kit (Qiagen, Hilden, Germany), with modifications to the lysis step to optimise DNA recovery from gram-positive bacteria. Specifically, the swab head was vortexed in 180 μL Buffer ATL for two minutes to dislodge bacterial cells. The swab was then removed with sterile forceps and 20 μL Proteinase K was added. The mixture was incubated at 56 °C for ∼18 h with light agitation (600 rpm). We used 30 μL UltraPure DNase/RNase-Free Water (Invitrogen, USA) for elution (DNA concentrations available in Supplementary Table 1: (see section on Supplementary materials given at the end of the article)). We included unused swabs as negative controls in each batch of extractions and quantified them to check for contamination. DNA from animal swabs were submitted to the Ramaciotti Centre for Genomics (University of New South Wales, Australia) for amplification using primers 341F (5′-CCTACGGGNGGCWGCAG-3′) and 805R (5′-GACTACHVGGGTATCTAATCC-3′) (Klindworth *et al.* 2012), targeting the V3-V4 regions of the bacterial 16S rRNA gene. All samples were uniquely barcoded, and amplicon libraries were sequenced on an Illumina MiSeq 2000 (Illumina, USA) using a 2 × 300 bp kit. A negative no-template control and positive ZymoBIOMICS Microbial Community DNA Standard (Zymo Research, USA) were included for quality control.

### Data processing and statistical analysis

We processed sequences using the Mothur (https://mothur.org; v.1.43.0) pipeline, implemented in Galaxy, according to the MiSeq SOP recommendations for 16S rRNA paired-end reads (Schloss *et al.* 2009). We merged paired-end reads into contigs and aligned unique reads against the SILVA 16S database release 138.1 (Quast *et al.* 2013), removing all sequences that did not align to the V3-V4 16S region (SILVA alignment position 6,388–25,316). An average of 4.2% of sequences across all samples (min. = 0.8%, max. = 12.5%) were removed (Supplementary Table 2). We included a pre-clustering step to group sequences, allowing up to 4 bp differences between sequences in each group, for the 465 bp long V3-V4 region (Schloss *et al.* 2011). Chimeric sequences were identified and cleaned from the data using the UCHIME algorithm in Mothur (Edgar *et al.* 2011). We clustered representative sequences into operational taxonomic units (OTUs) at genetic distances of 0.03, based on the naïve Bayesian classifier (Wang *et al.* 2007). Taxonomy was assigned based on the Ribosomal Database Project reference taxonomy (Cole *et al.* 2014). To control for variation in sequencing coverage, we rarefied the data by subsampling reads to the lowest sequencing depth in our sample set (14,138 reads for male samples and 16,212 reads for female samples). To assess sequencing adequacy, rarefaction curves were generated (Supplementary Fig. 1) and Good’s coverage scores were calculated (estimates ranged from 98.1 to 99.2%).

To address our first aim, we calculated alpha diversity of male external skin and internal pouch samples in Mothur using the command *summary.single* with two community richness calculators, observed number of OTUs and the Chao1 index (Chao 1984), and one community richness and evenness metric, the Shannon index (Shannon 1948). In a community, richness refers to the number of species, evenness to their relative abundances and diversity to both richness and evenness. Observed number of OTUs is a simple count of the number of OTUs in each sample, while the Chao1 index estimates species richness by counting the number of species and emphasising rare taxa that only appear once or twice (Chao 1984). The Shannon index is a measure of species diversity that takes into account both evenness and species richness (Shannon 1948). To test the effect of pregnancy and sampling location on each alpha diversity metric, we fitted a linear mixed-effect model using the lmer function in the package lme4 (Bates *et al.* 2015) using a Gaussian error function. We tested this model using the lmerTest package (Kuznetsova *et al.* 2017). Fixed factors included ‘sampling location’ (with two levels: skin (=0) and pouch (=1)) and ‘reproductive status’ (with two levels: non-pregnant (=0) and pregnant (=1)), with a random factor of ‘animal’ to account for repeated measures, as one skin and one pouch sample were taken from the same individual. Although the sample size for each group (*n* = 5) did not provide sufficient statistical power to formally include an interaction term in our main effects analysis, we conducted pairwise comparisons to test the differences between our four experimental groups using the estimated marginal means with Tukey’s HSD adjustment in the emmeans package (https://cran.r-project.org/web/packages/emmeans/index.html). To analyse and visualise beta diversity, we generated a Bray–Curtis dissimilarity matrix using dist.shared (Bray & Curtis 1957), to estimate differences in diversity and community structure between different male microbiomes. We tested main factor effects using PERMANOVA (999 permutations), using the adonis function in the vegan package (https://cran.r-project.org/web/packages/vegan/index.html). We tested differences between experimental groups using the pairwise.adonis2 function in the pairwiseAdonis package (https://github.com/pmartinezarbizu/pairwiseAdonis). Taxonomic composition of male microbiomes was explored by joining the taxonomic profile (tax.summary output) and summary of per-sample OTU occurrence (mothur.shared output) data frames to calculate relative abundances of taxa in each experimental group.

To address our second aim, we compared taxonomic profiles between non-pregnant and pregnant pouch microbiomes. We identified taxa exclusive to the pregnant pouch by searching for taxa that were absent in all non-pregnant pouch samples but present in most (at least 60%, 3 out of 5) of the pregnant pouch samples. A 60% cutoff allowed us to include more taxa in the core microbiome, given our small sample size. We then determined whether these taxa were present in any female egg samples (egg surface and egg surface plus egg contents). For each taxon present, we recorded the number of egg surface samples and egg surface plus egg contents samples it occurred in. To explore taxonomic composition of egg microbiomes, we calculated the relative abundance of bacterial taxa within each experimental group.

## Results

### Aim 1: male non-pregnant and pregnant external skin and internal pouch microbiomes

#### Alpha and beta diversity

**Figure 2:**
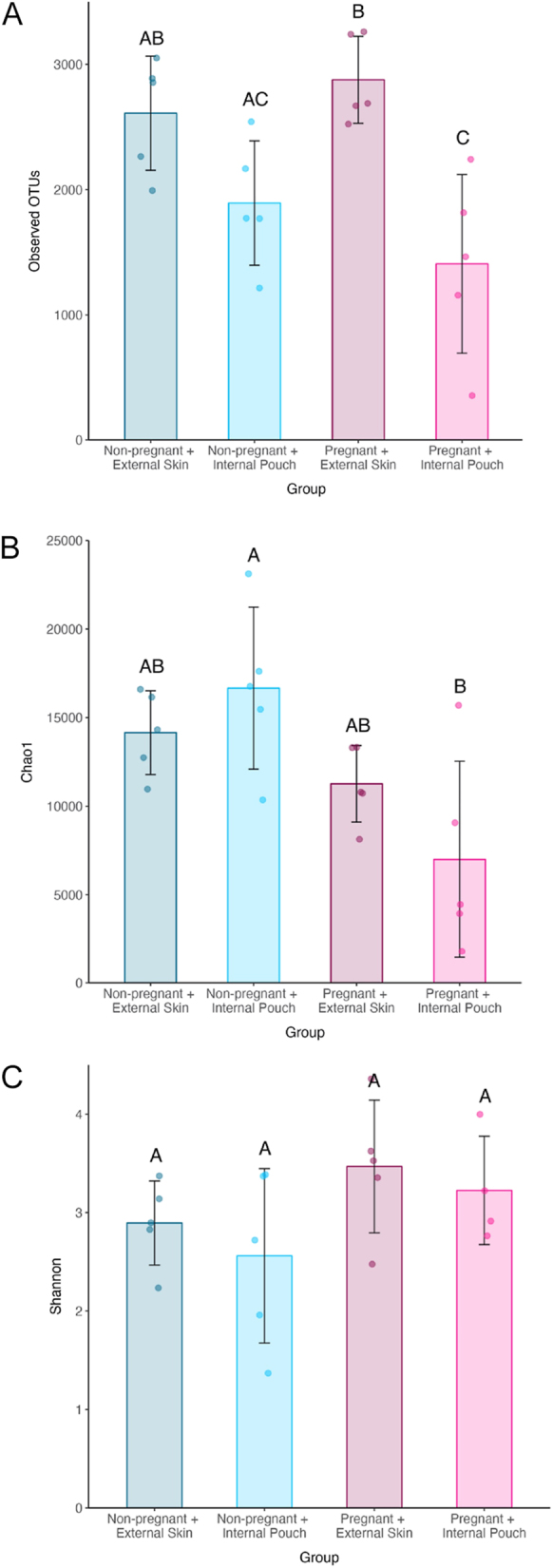
Mean richness measured by (A) observed OTUs, (B) Chao1 index, and mean diversity measured by (C) Shannon’s index, of four experimental group microbiomes in *Hippocampus abdominalis* males: non-pregnant external skin (*n* = 5), non-pregnant internal pouch (*n* = 5), pregnant external skin (*n* = 5) and pregnant internal pouch (*n* = 5). Error bars represent ±standard deviation about the mean. Lettering denotes significant differences.

The mean observed OTUs of external skin microbiomes was significantly higher than that of internal pouch microbiomes, while the mean observed OTUs of non-pregnant microbiomes was no different from that in pregnant microbiomes (Table 1, Fig. 2A). The pregnant internal pouch microbiome had the lowest number of observed OTUs out of all four groups (Fig. 2A). The mean Chao1 index of non-pregnant microbiomes was significantly higher than that of pregnant microbiomes, while the mean Chao1 index of external skin microbiomes was no different to that of internal pouch microbiomes (Table 1, Fig. 2B). The pregnant internal pouch microbiome had the lowest Chao1 index out of all four groups (Fig. 2B). As compared to the non-pregnant internal pouch, the pregnant internal pouch microbiome had a significantly lower Chao1 index (Table 1). The mean Shannon’s index of external skin microbiomes was no different to that of internal pouch microbiomes, and the mean Shannon’s index of non-pregnant microbiomes was also no different to that of pregnant microbiomes (Table 1, Fig. 2C). The pregnant internal pouch microbiome had a similar Shannon’s index compared to the other three groups (Fig. 2C).

**Table 1:** Results from linear mixed-effect models on effects of ‘reproductive status’ (non-pregnant vs pregnant) and ‘sampling location’ (skin vs pouch) on three measures of alpha diversity in *Hippocampus abdominalis* males. (A) Random term ‘animal’ (individual) was included to account for repeated measures (i.e., skin and pouch specimens taken from the same individual, see the section on Methods). Also shown are pairwise comparisons between microbiomes of the four male experimental groups: non-pregnant external skin (*n* = 5), non-pregnant internal pouch (*n* = 5), pregnant external skin (*n* = 5) and pregnant internal pouch (*n* = 5).

Factor	Estimate	SE	df	*t* value	*P* value (>|t|)
Observed OTUs					
Main effects					
Intercept	2,798.4	210.8	17.0	13.28	2.11 × 10^−10^***
Sampling location (pouch)^†^	−1,092.9	243.4	17.0	−4.49	2.32 × 10^−4^***
Reproductive status (pregnant)^‡^	−109.5	243.4	17.0	−0.45	0.658
Pairwise experimental group comparisons					
Non-pregnant skin – non-pregnant pouch	717	326	8.0	2.20	0.204
Non-pregnant skin – pregnant skin	−267	329	16	−0.81	0.849
Non-pregnant skin – pregnant pouch	1,202	329	16	3.66	0.010*
Non-pregnant pouch – pregnant skin	−983	329	16	−2.99	0.039*
Non-pregnant pouch – pregnant pouch	486	329	16	1.48	0.474
Pregnant skin – pregnant pouch	1,469	326	8.0	4.50	8.70 × 10^−3^**
Chao1 index					
Main effects					
Intercept	15,846.0	1,658.3	14.2	9.56	1.41 × 10^−7^***
Sampling location (pouch)	−879.4	1845.8	9.0	−0.48	0.645
Reproductive status (pregnant)	−6,279.2	1948.4	8.0	−3.22	1.22 × 10^−2^*
Pairwise experimental group comparisons					
Non-pregnant skin – non-pregnant pouch	−2,513	2,188	8.0	−1.15	0.672
Non-pregnant skin – pregnant skin	2,887	2,488	15.2	1.16	0.660
Non-pregnant skin – pregnant pouch	7,159	2,488	15.2	2.88	0.050
Non-pregnant pouch – pregnant skin	5,400	2,488	15.2	2.17	0.176
Non-pregnant pouch – pregnant pouch	9,672	2,488	15.2	3.89	6.90 × 10^−3^**
Pregnant skin – pregnant pouch	4,272	2,188	8.0	1.95	0.281
Shannon index					
Main effects					
Intercept	2.805	0.282	12.4	9.94	2.90 × 10^−7^***
Sampling location (pouch)	−0.156	0.268	9.0	−0.58	0.576
Reproductive status (pregnant)	0.752	0.351	8.0	2.14	6.47 × 10^−2^
Pairwise experimental group comparisons					
Non-pregnant skin – non-pregnant pouch	0.3336	0.392	8.0	0.852	0.829
Non-pregnant skin – pregnant skin	−0.5738	0.447	15.2	−1.283	0.587
Non-pregnant skin – pregnant pouch	−0.5962	0.447	15.2	−1.333	0.557
Non-pregnant pouch – pregnant skin	−0.9074	0.447	15.2	−2.029	0.221
Non-pregnant pouch – pregnant pouch	−0.9298	0.447	15.2	−2.079	0.204
Pregnant skin – pregnant pouch	−0.0225	0.392	8.0	−0.057	1.000

*** *P* (>|t|) < 0.001.

** *P* (>|t|) < 0.01.

* *P* (>|t|) < 0.05.

^†^ Reference category: external skin.

^‡^ Reference category: non-pregnant.

DF, degrees of freedom.

The three alpha diversity statistics revealed differing patterns; while only sampling location significantly affected the mean observed OTUs, only reproductive status significantly affected the Chao1 index of microbiomes. The Shannon’s index was not significantly affected by either of the two tested factors. While the number of observed OTUs was relatively low in the non-pregnant internal pouch, it did have a high Chao1 index, indicating that it harboured a high number of OTUs that appeared only once or twice. In the pregnant pouch, despite its low richness with both the observed OTUs and Chao1 index metrics, it had a similar Shannon’s diversity index to that of the other groups. As Shannon’s diversity accounts for both richness of species and evenness between species, this result indicates that the pregnant pouch microbiome had a highly even community relative to the other groups in the study.

**Figure 3:**
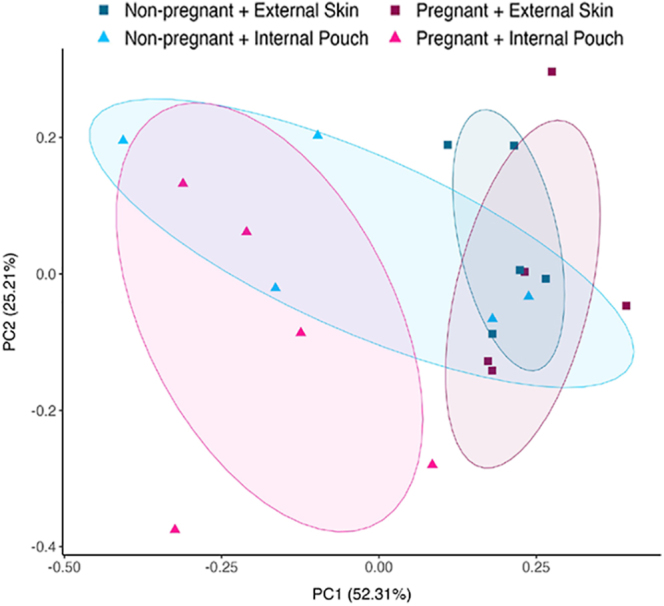
Principal coordinates analysis (PCoA) plot based on the Bray–Curtis distance matrix, depicting beta diversity across the four male microbiomes in *Hippocampus abdominalis*: non-pregnant external skin (*n* = 5), non-pregnant internal pouch (*n* = 5), pregnant external skin (*n* = 5) and pregnant internal pouch (*n* = 5). Ellipses illustrate 70% spread around the centroids within each experimental group.

Beta diversity describes the differences in diversity and community structure of microbiomes between samples. Microbiome community structure was significantly different between external skin and internal pouch and also significantly different between non-pregnancy and pregnancy (Table 2, Fig. 3). The pregnant internal pouch microbiome is significantly different from the non-pregnant internal pouch and both the pregnant and non-pregnant external skin (Table 2, Fig. 3).

**Table 2:** Results from PERMANOVA and pairwise comparisons on Bray–Curtis distance matrix, with 999 permutations, representing the beta diversity between male *Hippocampus abdominalis* microbiomes: non-pregnant external skin (*n* = 5), non-pregnant internal pouch (*n* = 5), pregnant external skin (*n* = 5) and pregnant internal pouch (*n* = 5). The two factors ‘sampling location’ and ‘reproduction status’ were included in the model, and the strata term ‘animal’ (individual) was used to account for repeated measures taken from the same individual.

Term	df	Sum of squares	*R*^2^ value	*F* value	*P* (>F)
PERMANOVA					
Sampling location	1	0.591	0.138	3.133	2.00 × 10^−3^**
Reproductive status	1	0.475	0.111	2.516	2.00 × 10^−3^**
Residual	17	3.207	0.751		
Pairwise comparisons					
Non-pregnant skin – non-pregnant pouch	1	0.237	0.146	1.366	0.184
Residual	8	1.388	0.854		
Non-pregnant skin – pregnant skin	1	0.244	0.208	2.107	0.069
Residual	8	0.926	0.792		
Non-pregnant skin – pregnant pouch	1	0.736	0.329	3.926	0.014*
Residual	8	1.500	0.671		
Non-pregnant pouch – pregnant skin	1	0.330	0.181	1.769	0.043*
Residual	8	1.492	0.819		
Non-pregnant pouch – pregnant pouch	1	0.446	0.178	1.729	0.037*
Residual	8	2.065	0.822		
Pregnant skin – pregnant pouch	1	0.570	0.262	2.842	5.00 × 10^−3^**
Residual	8	1.603	0.738		

** *P* (>F) < 0.01.

* *P* (>F) < 0.05.

DF, degrees of freedom.

Samples within both the non-pregnant and pregnant external skin microbiomes were clustered tightly together (Fig. 3), indicating that external skin microbiome community structure does not change greatly with pregnancy. While the non-pregnant pouch shared some similarity to the external skin microbiomes, the pregnant pouch microbiomes were distinct from the external skin microbiomes (Fig. 3).

#### Taxonomic composition of the male skin and brood pouch microbiomes

**Figure 4:**
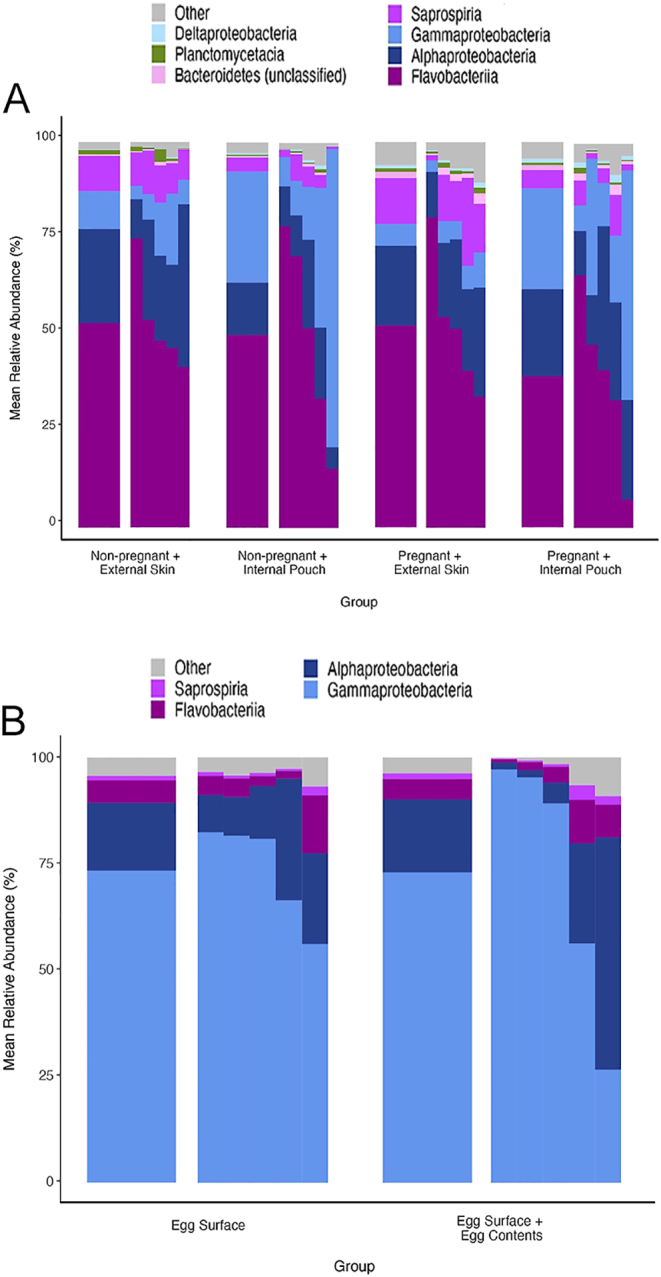
Taxonomic class composition of (A) male *Hippocampus abdominalis* microbiomes in four experimental groups (first bar): non-pregnant external skin (*n* = 5), non-pregnant internal pouch (*n* = 5), pregnant external skin (*n* = 5) and pregnant internal pouch (*n* = 5), and at individual sample level (second bar). (B) Female *H. abdominalis* microbiomes in two experimental groups (first bar): egg surface (*n* = 5) and egg surface plus egg contents (*n* = 5), and at individual sample level (second bar). Relative abundance of bacterial classes with over 1% abundance is shown.

Male skin and brood pouch microbiomes were dominated by the phyla Bacteroidetes (66.0%) and Proteobacteria (50.7%). The most abundant classes observed were Flavobacteriia (53.1%), Gammaproteobacteria (28.8%), Alphaproteobacteria (24.5%) and Saprospiria (11.9%) (Fig. 4A).

While we did not conduct statistical tests on taxonomic group abundance, the class Gammaproteobacteria appeared substantially more abundant in internal pouch microbiomes than on the external skin (Fig. 4A). At a finer taxonomic resolution, Gammaproteobacteria in the two internal pouch groups consisted of different genera. Within class Gammaproteobacteria, the non-pregnant internal pouch had a much higher relative abundance of the genera *Vibrio* (16.9%) and *Oceaniserpentilla* (4.35%) than in the three other male microbiomes (where abundances of both were <1.00%). In the pregnant internal pouch, the genera *Acinetobacter* (9.46%) and *Pseudomonas* (8.06%) occurred in high relative abundance as compared to the other male microbiomes (where abundances of both were <1.00%). The class Saprospiria was less abundant in the internal pouch microbiomes than on the external skin (Fig. 4A), with this difference mostly attributed to a lower relative abundance of an unclassified taxon in the order Saprospirales in the non-pregnant internal pouch (3.16%) and pregnant internal pouch (3.68%) as compared to the non-pregnant external skin (8.63%) and pregnant external skin (7.85%). The relative abundance of family *Flavobacteriaceae*, in class Flavobacteriia, was lowest in the pregnant internal pouch (38.3%) as compared to the non-pregnant internal pouch (49.6%), non-pregnant external skin (52.9%) and pregnant external skin (51.6%).

### Aim 2: bacterial taxa exclusive to the male pregnant pouch and overlap with female eggs

We identified 13 taxa that were not present in the non-pregnant pouch microbiome but were present in most (60%) of the pregnant pouch microbiome samples (Tables 3 and 4). Seven of the 13 taxa exclusive to the pregnant pouch, compared to the non-pregnant pouch, were also present in egg samples (Table 5). The pregnant pouch taxa *Cellulophaga*, *Kangiella*, *Maritalea*, *Reyranella*, Rhizobiaceae (unclassified genus) and Oligoflexales (unclassified family and genus) were not present in any egg samples (Table 5). There were no taxa exclusive to just egg surface plus egg contents samples.

**Table 3:** Taxonomic classification of 13 bacterial taxa exclusive to the male *Hippocampus abdominalis* pregnant brood pouch microbiome in comparison to the non-pregnant pouch. The lowest level of taxonomic classification of each taxon is shown in bold.

Phylum	Class	Order	Family	Genus
Bacteroidetes	Chitinophagia	Chitinophagales	Chitinophagaceae	***Sediminibacterium*** *
	Flavobacteriia	Flavobacteriales	Flavobacteriaceae	***Cellulophaga***
	Saprospiria	Saprospirales	Lewinellaceae	***Flavilitoribacter*** *
Planctomycetes	Planctomycetacia	Pirellulales	Lacipirellulaceae	***Bythopirellula*** *
Proteobacteria	Alphaproteobacteria	Parvularculales	Parvularculaceae	***Marinicaulis*** *
		Rhizobiales	Devosiaceae	***Maritalea***
			**Rhizobiaceae**	(Unclassified)
		Rhodobacterales	Rhodobacteraceae	***Roseivivax*** *
		Rhodospirillales	Reyranellaceae	***Reyranella***
	Betaproteobacteria	**Burkholderiales** *	(Unclassified)	(Unclassified)
	Deltaproteobacteria	Desulfobacterales	**Desulfobacteraceae** *	(Unclassified)
	Gammaproteobacteria	Oceanospirillales	Kangiellaceae	***Kangiella***
	Oligoflexia	**Oligoflexales**	(Unclassified)	(Unclassified)

* Taxa that were present in female *Hippocampus abdominalis* egg microbiomes.

**Table 4:** Description of microbial characteristics, habitat and ecology of bacteria exclusive to the male *Hippocampus abdominalis* pregnant brood pouch microbiome (*n =* 5) and not present in non-pregnant brood pouch samples (*n =* 5). The lowest taxonomic classification is used for identification of each bacterial taxon.

Taxon	Description of microbial characteristics	Habitat	Ecology
*Sediminibacterium*	Gram-negative rods. Strictly aerobic to facultatively anaerobic (Kim *et al.* 2013b, 2016). Bacteria are motile by gliding	Sediments and soil in or around aquatic environments, including from fishbowls (*Sediminibacterium aquarii*) (Kim *et al.* 2013b, 2016, Sethuraman *et al.* 2022).	Putative commensalism with algae (Sethuraman *et al.* 2022)
*Cellulophaga*	Gram-negative, gliding, aerobic rods forming iridescent colonies (Chapelais-Baron *et al.* 2017). Antifouling and algicidal (Skerratt *et al.* 2002)	Associated with marine algae and isolated from sediments, marine animals and seaweeds (Lafleur *et al.* 2015, Chapelais-Baron *et al.* 2017)	Inhibits *Pseudomonas aeruginosa* growth (Lafleur *et al.* 2015)
*Flavilitoribacter*	Rod-shaped (García-López *et al.* 2019)	*F. nigricans* from beach sand (García-López *et al.* 2019)	Unknown
*Bythopirellula*	Gram-negative, oval to pear-shaped bacteria that bud (Storesund & Øvreås 2013, Wiegand *et al.* 2020)	*Bythopirellula goksoyri* is from deep sea iron hydroxide deposits (Storesund & Øvreås 2013) and *Bythopirellula polymerisocia* from a riverbank (Wiegand *et al.* 2020)	Unknown
*Marinicaulis*	Gram-negative, aerobic or facultatively anaerobic motile rods (Yu *et al.* 2018, Wang *et al.* 2019)	*M. aureus* and *M. flavus* both isolated from seawater (Yu *et al.* 2018, Wang *et al.* 2019)	Unknown
*Maritalea*	Gram-negative, motile, strictly aerobic rods with flagella (Hwang *et al.* 2009, Fukui *et al.* 2012)	Found from coastal seawater and in marine plankton and algae cultures (Hwang *et al.* 2009, Fukui *et al.* 2012)	Unknown
*Roseivivax*	Gram-negative, aerobic, motile rods with subpolar flagella (Suzuki *et al.* 1999, Chen *et al.* 2012)	Found on charophyte algae, epiphytes (Suzuki *et al.* 1999) and coral (Chen *et al.* 2012)	Unknown
*Reyranella*	Gram-negative, non-motile rods with aerobic growth (Lee *et al.* 2017)	Both aquatic sources (rivers and cooling towers) (Pagnier *et al.* 2011) and terrestrial sources (forest and agricultural soil) (Kim *et al.* 2013a, Lee *et al.* 2017)	*Reyranella massiliensis* is an intra-cellular bacteria of amoeba (Pagnier *et al.* 2011)
*Kangiella*	Gram-negative, non-motile rods (Yoon *et al.* 2004, Lee *et al.* 2013), with enriched protein degradation ability (Wang *et al.* 2018)	From marine environments including marine organisms, coastal seawater, tidal flat sediments and deep-sea sediments (Lee *et al.* 2013, Wang *et al.* 2018)	Unknown
Desulfobacteraceae (family)	Gram-negative, anaerobic and morphologically varied (Galushko & Kuever 2020). Members metabolise sulphate to sulfide (Kuever 2014)	Found in various aquatic habitats including in freshwater, saline or hypersaline waters, in sediment or on aquatic organisms (Ahn *et al.* 2009, Kuever 2014)	Unknown
Rhizobiaceae (family)	A family of gram-negative rods, predominantly aerobic and highly heterogeneous (Carareto Alves *et al.* 2014)	Highly diverse, but mostly associated with soil and plants (Carareto Alves *et al.* 2014)	Facilitate nitrogen fixation in plants (Carareto Alves *et al.* 2014)
Burkholderiales (order)	Highly varied gram-negative bacteria. Strictly aerobic to facultatively anaerobic (Garrity *et al.* 2005)	Highly diverse habitats (Garrity *et al.* 2005)	Pathogenic and non-pathogenic (Garrity *et al.* 2005)
Oligoflexales (order)	Gram-negative, pleomorphic, obligately aerobic bacteria, with filamentous stages (Hahn *et al.* 2017)	*Oligoflexus tunisiensis* from desert sand (Nakai *et al.* 2014) and *Pseudobacteriovorax antillogorgiicola* from coastal coral (McCauley *et al.* 2015)	Unknown

**Table 5:** Presence of bacterial taxa exclusive to the male pregnant brood pouch compared to the non-pregnant pouch in female egg surface (ES; *n* = 5) and egg surface plus egg contents samples (ES+C; *n* = 5) in *Hippocampus abdominalis*.

Bacterial taxa	Presence in egg samples	
	**ES**	**ES+C**
*Sediminibacterium*	3/5	5/5
*Bythopirellula*	3/5	2/5
*Marinicaulis*	3/5	2/5
Burkholderiales (unclassified family, genus)	3/5	0/5
*Flavilitoribacter*	2/5	1/5
Desulfobacteraceae (unclassified genus)	1/5	1/5
*Roseivivax*	1/5	0/5
*Cellulophaga*	0/5	0/5
*Kangiella*	0/5	0/5
*Maritalea*	0/5	0/5
Oligoflexales (unclassified family, genus)	0/5	0/5
*Reyranella*	0/5	0/5
Rhizobiaceae (unclassified genus)	0/5	0/5

#### Taxonomic composition of female egg microbiomes

Egg microbiomes were largely dominated by the phylum Proteobacteria (91.2%), followed by Bacteroidetes (6.99%) and, at class level, were composed mostly of Gammaproteobacteria (73.4%), Alphaproteobacteria (17.2%) and Flavobacteriia (5.26%) (Fig. 4B).

Egg surface and egg surface plus egg contents microbiomes had very similar taxonomic compositions (Fig. 4B). Together, the egg microbiomes were dominated by the genus *Vibrio* (52.5%), followed by *Ruegaria* (9.96%), *Acinetobacter* (7.19%), an unclassified taxon in the class Gammaproteobacteria (5.96%) and *Pseudomonas* (4.12%). Like the male pregnant internal pouch, the female egg microbiomes harboured a relatively high abundance of the genera *Acinetobacter* and *Pseudomonas* compared to all other male microbiomes. *Vibrio* occurred in high relative abundance in both the non-pregnant internal pouch and egg microbiomes, although these taxonomic abundance differences were not explicitly tested.

## Discussion

Our study compared the male pregnant seahorse pouch microbiome with the non-pregnant pouch and external skin and identified bacterial taxa exclusive to the pregnant pouch, potentially derived from eggs. The pregnant pouch microbiome was compositionally unique, with a lower species richness but higher evenness as compared to the other male microbiomes. The abundance of *Vibrio* was high in the non-pregnant pouch but very low in the pregnant pouch. We also identified 13 bacterial taxa unique to the pregnant pouch and inferred that seven of these taxa were potentially derived from eggs, suggesting a maternal microbial contribution to the embryonic environment.

We determined that the male pregnant brood pouch harbours an internal microbiome distinct from the external skin and non-pregnant pouch. The brood pouch encloses after mating (Berglund & Rosenqvist 1990), which we postulate creates a barrier against external bacterial immigration, fostering an isolated microbial habitat. The microbial community shift from non-pregnancy to pregnancy may result from physiological changes. For instance, endocrine changes can mediate reproductive microbiome structure (Comizzoli *et al.* 2021). Glucocorticoids modulate the reproductive microbiome in other animals, including rhinoceros and humans (Amabebe & Anumba 2018, Antwis *et al.* 2019). Glucocorticoids also increase during seahorse pregnancy to stimulate pouch growth (Scobell & Mackenzie 2011). Sex hormones, including oestradiol and progesterone, also modify reproductive tissues by enhancing their epithelial receptivity to beneficial bacteria (Stumpf *et al.* 2013). The same hormones stimulate epithelium proliferation of pregnant seahorse pouch tissue (Oconer *et al.* 2003), which may contribute to the pouch microbiome shift with pregnancy we report here. Nutritional changes in the pouch likely also influence the microbiome. Pregnant syngnathid pouch fluid contains lipid-rich yolk from fragmented eggs (Linton & Soloff 1964, Ripley & Foran 2009). This increased nutrient density may enhance the dominance of saprophytic bacteria that thrive by metabolising organic compounds including lipids and amino acids. *Acinetobacter* and *Pseudomonas* are such saprophytes (Cousin 1999, Kämpfer 2014), explaining why these taxa were highly abundant in nutrient-rich eggs and inside the pregnant pouch in our study. The salinity inside the pouch changes to match external marine conditions in late pregnancy (Oconer *et al.* 2006), which may also alter the resident microbiome. Our use of artificial seawater might have cultivated a slightly different pregnant pouch microbiome compared to wild seahorses experiencing natural environmental salinity fluctuations. There are multiple plausible physiological mechanisms that may contribute to the observed shift in brood pouch microbiome and further investigations into these relationships will help to clarify how the seahorse gestational microbiome is formed. We note that our swabbing approach samples the overall gestational environment but does not allow differentiation of the pregnant pouch versus embryonic microbiomes. Future studies aiming to isolate taxa tightly associated with the embryos or pouch could use surface sterilised whole embryos or dissected pouch tissue directly.

We observed that the male pregnant pouch microbiome exhibits low species richness, high species evenness and low *Vibrio* abundance, patterns which suggest that the pouch environment is pathogen limited. First, the pregnant pouch has a low richness, especially of rare taxa, compared to the non-pregnant pouch. A low microbial richness in the gestational environment is also evident in marsupial pouches, such as those of the tammar wallaby (*Macropus eugenii*) and the southern hairy-nosed wombat (*Lasiorhinus latifrons*) to protect developing young (Old & Deane 1998, Weiss *et al.* 2021). Like seahorse embryos, marsupial young spend a large proportion of their development inside a pouch. In marsupials, antimicrobial secretions help form this protective environment (Weiss *et al.* 2021). Similarly, antimicrobial peptides are also secreted into the seahorse pouch (Melamed *et al.* 2005), and pouch-expressed genes encoding other immune factors, such as cytokines and pathogen recognition receptors, are also upregulated during pregnancy (Wu *et al.* 2021, Jiang *et al.* 2022). These immune factors may reduce pathogenic challenges inside the seahorse pouch and reduce embryo mortality, a hypothesis congruent with our finding of a low occurrence of rare species in the pregnant pouch microbiome. Our study used captive laboratory-reared seahorses fed frozen food. While captive and wild fish may have a similar core microbiome (Roeselers *et al.* 2011), captivity may limit their exposure to rare micro-organisms compared to wild counterparts inhabiting diverse marine microbiomes and consuming varied diets (Clavere-Graciette *et al.* 2022, Ortega-Kindica *et al.* 2024). Future studies on wild seahorses are warranted to confirm whether these patterns persist in natural marine environments.

We also found that the pregnant pouch microbiome maintained high species evenness (and therefore diversity), which may confer pathogen colonisation resistance. Laboratory-controlled studies of bacterial communities have shown that increased evenness enhances resistance to invasion by alien species (De Roy *et al.* 2013). From a marine perspective, in oysters, surface microbiomes of higher evenness exhibit resistance against the pathogenic ostreid herpesvirus (Clerissi *et al.* 2020). In the broad-nosed pipefish (*Syngnathus typhle*), a highly diverse internal pouch microbiome is thought to protect embryos (Tanger *et al.* 2024). The high level of evenness observed in the pregnant seahorse pouch microbiome in our study may similarly suggest resilience against pathogenic invasion. Finally, while *Vibrio* abundance was high in the non-pregnant pouch (and eggs), it was very low in the pregnant pouch (and external skin). *Vibrio* comprises a large number of opportunistic marine pathogens, including *V. harveyi*, *V. alginolyticus* and *V. splendidus*, which are linked to diseases of the external tissues, such as the skin and gills, in seahorses (Alcaide *et al.* 2001, Balcázar *et al.* 2010, Binh *et al.* 2016). In our study, which used healthy (captive) seahorses, *Vibrio* abundance was indeed low on the external skin. It is notable that *Vibrio* is limited in the pregnant pouch, despite its high abundance in the non-pregnant pouch and further enrichment with *Vibrio* from the eggs, indicating that the pregnant pouch environment may actively reduce *Vibrio* abundance. Alternatively, microbe–microbe interactions could limit *Vibrio* proliferation, although this hypothesis requires further investigation into the pouch microbiome’s community dynamics. The low taxonomic resolution of *Vibrio* in our study is a constraint and future research should examine species and strain level patterns to enable more concrete biological inferences. Still, our findings highlight an interesting starting point to test whether the pregnant pouch microbiome reduces the microbial burden for offspring. Functional studies are needed to resolve the adaptive significance of the pregnant pouch microbiome. Testing wild seahorses could validate whether low *Vibrio* abundance occurs in the pouch under natural conditions. In addition, examining immune gene expression in pregnant pouch tissue in both captive and wild seahorses could clarify whether low *Vibrio* abundance correlates with heightened local immune activity.

While *Vibrio* abundance was low in the male pregnant pouch, this genus dominated female egg microbiomes. Although some *Vibrio* species are associated with seahorse skin disease, which may explain the genus’s low abundance in the pregnant pouch, several *Vibrio* species are commensal and abundant in the seahorse gut microbiome (Balcazar *et al.* 2010, Tanu *et al.* 2012, Wang *et al.* 2020). It is possible that gut commensal *Vibrio* species are contained inside the egg coat, enabling ingestion and early gut colonisation in offspring, while the pregnant pouch environment surrounding the eggs suppresses the abundance of *Vibrio* skin pathogens, to prevent embryo skin infections post-hatching. A study of the broad-nosed pipefish (*Syngnathus typhle*) tracked the source of micro-organisms that colonise offspring, and posited that the paternal brooding environment mostly influences the offspring external microbiome, while maternal transmission via eggs is responsible for internal colonisation of the offspring gut (Tanger *et al.* 2024). Our results are consistent with this concept, suggesting a similar mechanism in seahorses. To test this hypothesis, the embryo microbiome before and after hatching from the egg coat, which occurs within the pouch (Sommer *et al.* 2012), should be compared to reveal whether gut commensals are abundant before hatching, and whether skin commensals are enriched and/or skin pathogens are reduced after hatching. Comparison of surface sterilised embryos versus unsterilised embryos would also enable differentiation between the internal (gut) and external (skin) embryonic microbiomes (Tanger *et al.* 2024).

We identified the genera *Cellulophaga* and *Sediminibacterium* in the pregnant pouch (but not the non-pregnant pouch), the latter taxon being potentially maternally derived. These taxa are interesting due to their antimicrobial characteristics. *Cellulophaga* inhibits pathogenic *Pseudomonas* spp., including *Pseudomonas aeruginosa*, by disrupting virulent colony formation (Lafleur *et al.* 2015, Chapelais-Baron *et al.* 2017). While *Pseudomonas aeruginosa* pathogenicity is more widely acknowledged in freshwater fish, it is present in coastal seawater, where it can infect marine species (Kimata *et al.* 2004, El-Dakroury *et al.* 2020). The presence of *Cellulophaga* spp. in the pregnant pouch may protect embryos from such infections. While we recognised *Pseudomonas* to be relatively abundant in the pregnant pouch, potentially due to its ability to proliferate in nutrient-rich environments, the species in this genus are diverse, and the lack of taxonomic resolution here prevents detailed inferences about its interaction with *Cellulophaga*. *Sediminibacterium*, which occurred in a high proportion of egg samples, belongs to the family Chitinophagaceae, which is known for its ability to inhibit fungal growth by degrading chitin, a fungal cell wall component (Rosenberg 2014), and secreting antibiotic compounds against gram-positive pathogens (Beckmann *et al.* 2017). Chitinophagaceae presence is protective in some fish: increases in Chitinophagaceae abundance in sea bass (*Lateolabrax maculatus*) gut and three-spined stickleback (*Gasterosteus aculeatus*) water are associated with host disease resistance (Deng *et al.* 2021, Fuess *et al.* 2021). In our study, *Sediminibacterium* may share these microbicidal features, thereby protecting embryos. That *Sediminibacterium* may be transmitted to the pouch via the eggs demonstrates a potential maternal contribution to embryo protection.

To the best of our knowledge, this is the first study to explore the microbiome inside the pregnant male seahorse brood pouch. We found that the pregnant pouch harbours a distinct microbiome, compositionally different from the male non-pregnant pouch and external skin. The microbiome possibly attenuates the pathogenicity of the gestational environment, as the abundance of the genus *Vibrio* is greatly reduced. The low richness of rare bacteria and high diversity of the pregnant pouch microbiome suggest resistance to pathogen colonisation, possibly providing immunological protection for offspring. The eggs may also supplement the pouch with beneficial bacteria, such as *Sediminibacterium*, and prime offspring with gut commensals. Our characterisation of the seahorse brood pouch microbiome provides a valuable foundation for further investigation of the function of the gestational microbiome in male pregnancy.

## Supplementary materials



## Declaration of interest

The authors declare that there is no conflict of interest that could be perceived as prejudicing the impartiality of the work reported.

## Funding

This work was supported by a University of Sydney Research Accelerator (SOAR) Prize and Australia and Pacific Science Foundationhttps://doi.org/10.13039/501100001037 Funding to CMW.

## Author contribution statement

JW, CMW and CEG conceived and designed the study. JW performed the experiments and analysed data with guidance from CEG, CMW and ZS. JW, CMW and CEG wrote the manuscript, with editing from ZS. CMW obtained funding for this study. All authors provided feedback throughout the study to produce the data, analyses and final manuscript.

## Data availability

All raw 16S amplicon reads are available in the NCBI SRA database under accession number PRJNA1227541.
