# Supplementary material for: Changes to the reproductive microbiome of the brood pouch during male pregnancy in seahorses (Hippocampus abdominalis)
Source: Reproduction. 2025 Mar 10;169(4):e240159. doi: 10.1530/REP-24-0159 (PMC11906128; doi:10.1530/REP-24-0159)
Supplement: Supplementary file 1 [file supplementary_materials.pdf]

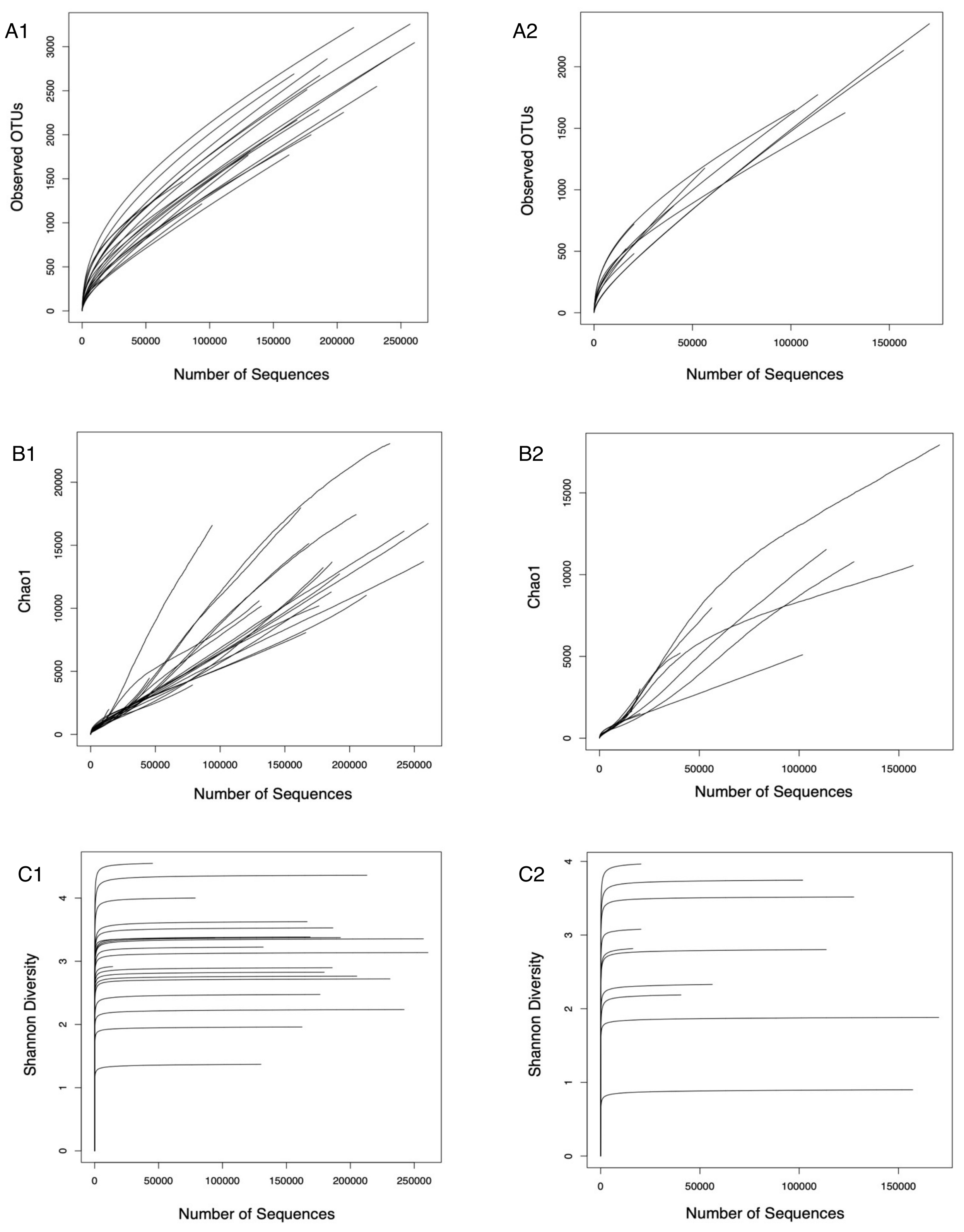

Supplementary Figure 1: Rarefaction curves demonstrating the relationship between number of sequences (sequencing depth) and A) Observed OTUs, B) Chao1 index, and C) Shannon index, for *Hippocampus abdominalis* 1) male external skin and internal brood pouch, and 2) female egg samples.

Supplementary Table 1: DNA concentrations of male and female *Hippocampus abdominalis* microbiome samples, quantified by NanoDrop™ One Spectrophotometer.

Sex of Animal	Experimental Group	Sample Name	DNA concentration	A260/280
Female	Egg Surface	Hab2155F-IE	11.89	1.84
Female	Egg Surface + Egg Contents	Hab2155F-OE	8.22	1.69
Female	Egg Surface	Hab2156F-IE	34.63	1.97
Female	Egg Surface + Egg Contents	Hab2156F-OE	22.47	2.04
Female	Egg Surface	Hab2158F-IE	38.05	2.05
Female	Egg Surface + Egg Contents	Hab2158F-OE	30.99	1.97
Female	Egg Surface	Hab2159F-IE	29.57	1.98
Female	Egg Surface + Egg Contents	Hab2159F-OE	19.67	1.91
Female	Egg Surface	Hab2160F-IE	96.91	2.10
Female	Egg Surface + Egg Contents	Hab2160F-OE	62.98	2.10
Male	Pregnant + Internal Pouch	Hab2130MP-IP	3.80	1.64
Male	Pregnant + External Skin	Hab2130MP-ES	7.16	1.92
Male	Pregnant + Internal Pouch	Hab2131MP-IP	8.85	1.81
Male	Pregnant + External Skin	Hab2131MP-ES	7.60	1.95
Male	Pregnant + Internal Pouch	Hab2135MP-IP	124.45	2.11
Male	Pregnant + External Skin	Hab2135MP-ES	10.33	2.10
Male	Pregnant + Internal Pouch	Hab2136MP-IP	84.67	2.11
Male	Pregnant + External Skin	Hab2136MP-ES	23.22	2.11
Male	Pregnant + Internal Pouch	Hab2137MP-IP	10.58	1.78
Male	Pregnant + External Skin	Hab2137MP-ES	19.44	2.00
Male	Non-pregnant + Internal Pouch	Hab2149MNP-IP	9.73	1.92
Male	Non-pregnant + External Skin	Hab2149MNP-ES	4.83	2.09
Male	Non-pregnant + Internal Pouch	Hab2150MNP-IP	14.83	1.91
Male	Non-pregnant + External Skin	Hab2150MNP-ES	2.37	2.07
Male	Non-pregnant + Internal Pouch	Hab2152MNP-IP	4.68	1.87
Male	Non-pregnant + External Skin	Hab2152MNP-ES	9.04	2.06
Male	Non-pregnant + Internal Pouch	Hab2153MNP-IP	4.54	1.69
Male	Non-pregnant + External Skin	Hab2153MNP-ES	13.69	1.99
Male	Non-pregnant + Internal Pouch	Hab2154MNP-IP	3.99	1.90
Male	Non-pregnant + External Skin	Hab2154MNP-ES	48.98	2.12

Supplementary Table 2: Number of sequences before and after sequence alignment in female and male *Hippocampus abdominalis* microbiome samples. Percentage of sequences removed per sample in this sequence processing step is shown.

Sex of Animal	Experimental Group	Sample Name	No. Sequences Pre-Alignment	No. Sequences Post-Alignment	Percentage Sequences Removed
Female	Egg Surface	Hab2155F-IE	61068	57694	5.5%
Female	Egg Surface + Egg Contents	Hab2155F-OE	41929	39745	5.2%
Female	Egg Surface	Hab2156F-IE	10335	9046	12.5%
Female	Egg Surface + Egg Contents	Hab2156F-OE	7967	7638	4.1%
Female	Egg Surface	Hab2158F-IE	41073	40369	1.7%
Female	Egg Surface + Egg Contents	Hab2158F-OE	46394	43450	6.3%
Female	Egg Surface	Hab2159F-IE	20391	18457	9.5%
Female	Egg Surface + Egg Contents	Hab2159F-OE	5857	5235	10.6%
Female	Egg Surface	Hab2160F-IE	41788	40172	3.9%
Female	Egg Surface + Egg Contents	Hab2160F-OE	13025	11696	10.2%
Male	Pregnant + Internal Pouch	Hab2130MP-IP	6394	6342	0.8%
Male	Pregnant + External Skin	Hab2130MP-ES	70225	69327	1.3%
Male	Pregnant + Internal Pouch	Hab2131MP-IP	20822	18874	9.4%
Male	Pregnant + External Skin	Hab2131MP-ES	94133	92825	1.4%
Male	Pregnant + Internal Pouch	Hab2135MP-IP	48394	45831	5.3%
Male	Pregnant + External Skin	Hab2135MP-ES	82804	81126	2.0%
Male	Pregnant + Internal Pouch	Hab2136MP-IP	27254	26070	4.3%
Male	Pregnant + External Skin	Hab2136MP-ES	57837	56662	2.0%
Male	Pregnant + Internal Pouch	Hab2137MP-IP	55853	54999	1.5%
Male	Pregnant + External Skin	Hab2137MP-ES	54753	53977	1.4%
Male	Non-pregnant + Internal Pouch	Hab2149MNP-IP	39440	38291	2.9%
Male	Non-pregnant + External Skin	Hab2149MNP-ES	51751	51070	1.3%
Male	Non-pregnant + Internal Pouch	Hab2150MNP-IP	41642	38355	7.9%
Male	Non-pregnant + External Skin	Hab2150MNP-ES	51427	50780	1.3%
Male	Non-pregnant + Internal Pouch	Hab2152MNP-IP	47350	45927	3.0%
Male	Non-pregnant + External Skin	Hab2152MNP-ES	61215	59952	2.1%
Male	Non-pregnant + Internal Pouch	Hab2153MNP-IP	28448	27446	3.5%
Male	Non-pregnant + External Skin	Hab2153MNP-ES	77337	75854	1.9%
Male	Non-pregnant + Internal Pouch	Hab2154MNP-IP	52979	51912	2.0%
Male	Non-pregnant + External Skin	Hab2154MNP-ES	58174	56995	2.0%
